# Physical function, psychological adjustment, and self-efficacy following sudden cardiac arrest and an initial implantable cardioverter defibrillator (ICD) in a social cognitive theory intervention: secondary analysis of a randomized control trial

**DOI:** 10.1186/s12872-022-02782-8

**Published:** 2022-08-10

**Authors:** Cynthia M. Dougherty, Ana Carolina Sauer Liberato, Megan M. Streur, Robert L. Burr, Ka Yee Kwan, Tao Zheng, Jon P. Auld, Elaine A. Thompson

**Affiliations:** 1 School of Nursing, Biobehavioral Nursing and Health Informatics, University of Washington, 1959 NE Pacific Street, Box 357266, Seattle, WA 98195 USA; 2 Evidera PPD, London, England, UK

**Keywords:** Sudden cardiac arrest, Intervention, Quality of life, Physical function, Symptoms, Psychological, Social cognitive theory, Implantable cardioverter defibrillator

## Abstract

**Background:**

Sudden cardiac arrest (SCA) survivorship results in unique issues in return to physical and psychological function. The purpose of the study was to compare recovery across the first year between SCA survivors and other arrhythmia patients who received a first-time implantable cardioverter defibrillator (ICD) for secondary prevention, participating in a social cognitive theory (SCT) intervention.

**Methods:**

168 (129 males, 39 females) who received an ICD for secondary prevention (SCA N = 65; other arrhythmia N = 103) were randomized to one of two study conditions: SCT intervention (N = 85) or usual care (N = 83). Outcomes were measured at baseline hospital discharge, 1, 3, 6, & 12 months: (1) Physical Function: Patient Concerns Assessment (PCA), SF-36 (PCS); (2) Psychological Adjustment: State Trait Anxiety (STAI), CES-D depression, SF-36 (MCS); (3) Self-Efficacy: Self-Efficacy (SCA-SE), Self-management Behaviors (SMB), Outcome Expectations (OE). Outcomes were compared over 12 months for intervention condition x ICD indication using general estimating equations.

**Results:**

Participants were Caucasian (89%), mean age 63.95 ± 12.3 years, EF% 33.95 ± 13.9, BMI 28.19 ± 6.2, and Charlson Index 4.27 ± 2.3. Physical symptoms (PCA) were higher over time for SCA survivors compared to the other arrhythmia group (*p* = 0.04), ICD shocks were lower in SCA survivors in the SCT intervention (*p* = 0.01); psychological adjustment (MCS) was significantly lower in SCA survivors in the SCT intervention over 6 months, which improved at 12 months (*p* = 0.05); outcome expectations (OE) were significantly lower for SCA survivors in the SCT intervention (*p* = 0.008).

**Conclusions:**

SCA survivors had greater number of physical symptoms, lower levels of mental health and outcome expectations over 12 months despite participation in a SCT intervention.

*Trial registration* Clinicaltrials.gov: NCT04462887.

## Background

Sudden cardiac arrest (SCA) is the cessation of cardiac mechanical activity that occurs without warning or with symptoms of < 1 h duration [1]. Age-adjusted mortality rates for SCA in the U.S. have declined from 138/100,000 in 1999 to 97.1/100,000 in 2017, thus impacting about 180,202 adults/year [2]. Approximately 70,000 individuals are discharged from the hospital each year after combined out-of-hospital and in-hospital cardiac arrest [3]. In the post-resuscitation period, the majority of survivors of SCA with cardiac disease will receive an implantable cardioverter defibrillator (ICD) as the mainstay of treatment.

At the time this study was conducted, the criteria for receipt of an ICD for secondary prevention of SCA included: (1) survivors of ventricular fibrillation (VF) or hemodynamically unstable ventricular tachycardia (VT), (2) structural heart disease with spontaneous sustained VT, (3) syncope with sustained VT or VF induced with electrophysiological study (EPS), or (4) non-sustained VT due to prior myocardial infarction (MI), left ventricular ejection fraction (LVEF)% ≤ 40%, and inducible VF or sustained VT at EPS [4]. Thus, survivors of SCA and those who receive an ICD are a heterogenous group, ranging from those who have no known cardiac disease to those who have complicated chronic cardiac conditions. SCA survivors present with unique cognitive, psychological, emotional, and functional needs as a consequence of resuscitation [5]. These unique needs are known to prevent return to work, resumption of normal daily activities, and performance of family and social roles, impacting overall quality of life. Evidence suggests that consequences of SCA survival can exist for long periods of time [1]. Interventions have been tested to address these unique needs and promote post-SCA adjustment [6, 7], but a consistent structured and comprehensive approach to assessment and rehabilitation after SCA is not available or part of routine post-arrest care. This study was conducted because we have RCT data from a large sample of secondary prevention patients, some of whom had experienced SCA. As well, the study addresses specific questions about the impact of a focused intervention on SCA recovery outcomes apart from other patients who get an ICD for complex arrhythmias.

The purpose of this study was to describe longitudinal change in physical and mental health over the first year post-implant for two distinct ICD secondary prevention groups: patients who experienced a sudden cardiac arrest (SCA) and those who received an ICD for other complex cardiac arrhythmias. We hypothesized that intervention effects across time would be moderated by a history of SCA [intervention x time x ICD indication]. This study fills a major gap in the scientific literature by identifying potential differences between SCA survivors and other ICD secondary prevention patients in response to an intervention program, designed to enhance quality of life and return to functional status. This study provides findings to address specific needs and experiences of SCA survivors, and describes outcomes comparable to the general secondary prevention ICD population.

## Methods

### Design

This secondary analysis is based on a longitudinal randomized clinical trial that tested the effects of a 2-month, combined education and telephone intervention, delivered by trained cardiovascular nurses, compared to usual care (UC) [6, 7]. The original RCT was conducted between 1999 and 2003, a time that preceded implantation of ICDs for primary prevention of SCA. The short-term [6] and longer term [7] primary outcomes of the intent-to treat results were reported previously. During this trial, the use of therapeutic hypothermia was not a standard of care for out-of-hospital cardiac arrest. All patients in this study received implantation of a transvenous lead ICD system. Measurements were collected the first week after hospital discharge, and at 1, 3, 6 and 12 months following hospitalization for the initial ICD implant. All research procedures were reviewed and approved by hospital Institutional Review Boards (IRBs) and the academic IRB prior to contact with potential participants. The protocol was conducted following relevant guidelines and regulations. Participants were identified during hospitalization by catheter laboratory nurses who worked in medical centers in the Pacific Northwest. All patients who agreed to speak with the study personnel were contacted by telephone the day after hospital discharge by the investigators who explained the study and obtained verbal consent to participate. Then, written informed consent and baseline measures were completed and participants were randomized to study condition (SCT intervention vs UC) using a random number generator program.

### Sample

Study participants (n = 168) included individuals who had experienced a first time out-of-hospital SCA or life-threatening ventricular arrhythmia requiring ICD implantation for secondary prevention based on established guidelines [8]. In this sample of 168, 65 patients had suffered an out-of-hospital cardiac arrest, achieved return of spontaneous circulation (ROSC), and were admitted and discharged from the hospital alive. The other 103 patients received an ICD for other secondary prevention reasons, including symptomatic and asymptomatic ventricular arrhythmias, with or without electrophysiologic (EPS) testing. Criteria also included the ability to read, speak and write English, having telephone access, and willingness to be followed for 1 year. Individuals were excluded from the study if they had significant clinical comorbidities that prevented their return home after hospitalization or if they were younger than 21 years of age. Confirmation of SCA and the need for ICD implantation were verified using medical records and EPS reports. All participants were screened at recruitment using the Short Blessed cognitive screening tool [9]. Short Blessed scores ≥ 10 indicated cognitive impairment too severe for participation. Two individuals were not eligible for study participation based on the Short Blessed criteria.

### Social cognitive theory (SCT) intervention

The SCT intervention consisted of two key components: (1) structured information (SI) provided in a booklet mailed to study participants, and (2) nursing telephone support (NTS) conducted by expert cardiovascular nurses. Expert cardiovascular nurses had a minimum of 5 years of experience as a cardiovascular nurse and had extensive training in the NTS protocol. Intervention nurses were part of the research team, not employed as staff nurses in the participating medical centers, and did not deliver usual care.

The intervention components were based on Bandura’s SCT [10] and previous research with survivors of SCA [11]. A complete description of the theoretical framework used [12] and the nursing intervention is published [13]. The nursing intervention was unlike any interventions currently used in clinical practice and was specifically designed to match the Domains of Concern previously validated with this population. The SI booklet, Sudden Cardiac Arrest: A Survivor’s Experience, contains two components: (1) a descriptive component including individual verbatim statements about experiences of others during the first year of recovery and (2) a management component outlining successful strategies (skills) used by others in dealing with issues in recovery. The purpose of the SI booklet was to describe the experiences one can expect to encounter during recovery after an ICD and offer suggestions for behavioral strategies to deal with the issues.

The NTS protocol was a telephone intervention delivered over 2 months subsequent to ICD implantation. The purposes of the NTS intervention were to (1) teach specific knowledge and behavioral skills needed to manage ICD recovery, (2) enhance self-confidence (self-efficacy) in one’s ability to deal with illness demands, and (3) reduce emotional arousal and anxiety. Telephone calls were designed to last approximately 15–20 min. Each call was carefully scripted to include the following elements: (1) check-in about current concerns, (2) assessment of the topic for the week, (3) review of common recovery experiences, (4) discussion of behavioral strategies for dealing with the topic for the week, (5) provision of positive feedback for strategies already working well, (6) anxiety reduction statements, (7) practice of new behaviors using role-playing and problem solving techniques, (8) summarization, (9) setting specific goals for the upcoming week, and (10) collaborating on a learning assignment for the subsequent week. Content of the nursing intervention included physical symptoms, activity progression and exercise, emotional reactions, ICD shocks, partner relationships, safety and maintenance of the ICD, and dealing with health care providers.

Intervention participants received study materials following completion of the baseline questionnaire. Participants were asked to read the SI booklet within the first week after hospital discharge and to refer to it during NTS calls. All intervention calls occurred during the first 2 months following ICD implantation. In addition to the NTS calls, intervention participants could access the intervention nurse during regular business hours via a toll free telephone Monday to Friday, or via pager after hours (24 h/day). Over a 2-year period, 16 (19%) of 84 individuals used the nurse pager a total of 18 times, the majority of whom called about the ICD (75%). The other 25% of calls were related to general medical topics.

Usual care participants received standard treatment from their health care providers. Usual care consisted of standardized hospital-based education about the ICD in the form of a booklet, videotape, or both. A pre-study program review revealed that the type and content of information provided during hospitalization were similar across the ten recruitment sites. Both groups were monitored for exposure to additional interventions, participation in educational or support groups, and counseling. In both groups, 97% reported receiving ICD device information and/or viewing an ICD related videotape during their hospitalization for ICD implantation. Both groups received outpatient follow-up clinic visits at times and frequency as designated by their health care providers.

### Measures

Study outcomes were measured 5 times over 12 months, starting with hospital discharge following ICD implant and then at 1, 3, 6, 12 months later. Unless otherwise noted, higher values denote higher levels of the measured construct; reported internal consistency values (Cronbach’s alpha, α) are based on the study sample.

**Table:** 

Outcome variable & measures	Description of measure
*Physical function*	
Patient concerns assessment (PCA) [14], Range 0–29	PCA is symptom checklist (α = 0.88) that measures physical symptoms and fears common in the first few months after ICD implantation
Short form health Survey (SF-12) [15], Range PCS 15–60, Range MCS 24–67	SF-12 is a 12-item measure of general health related to physical and mental health. Two component summary scales of physical (PCS, α = 0.94) and mental health (MCS, α = 0.94) are derived
Cardiac arrhythmias, Range 0–12	ICD shocks and heart rhythm stability assessed using interrogation reports from ICD de-vices during routine follow-up visits. The total number of ICD shocks received whether or not appropriate for the cardiac rhythm were recorded
*Psychological adjustment*	
Anxiety [16], Range 20–68	State-Trait Anxiety Inventory (STAI) measure of anxiety (α = 0.84) is used extensively in cardiovascular populations. Higher scores denote higher anxiety. This study used the state scale of the inventory only
Depression [17], Range 0–51	Center for Epidemiologic Studies Depression (CES-D)^16^ is a measure (α = 0.87) of depressive symptomatology in the general population with an emphasis on depressed mood
*Self-efficacy*	
Self-efficacy expectations [18], Range 2–10	Sudden cardiac arrest -self efficacy (SCA-SE) scale focuses on self-efficacy expectations about one’s ability to manage common problems after sudden cardiac arrest and an ICD (α = 0.93)
Self-management behaviors [18], Range 0–10	Sudden cardiac arrest behaviors (SCA-B) scale focuses on behaviors required to manage common problems after sudden cardiac arrest and an ICD (α = 0.89)
Outcome expectations [18], Range 2–5	Sudden cardiac arrest outcome expectations (SCA-OE) (α = 0.81) focus on perceived consequences of engaging in self-management behaviors after sudden cardiac arrest and an ICD

### Analysis

Initially, the distributional properties of the study variables, outlier cases, and missing data were characterized. As the proportion of missing data was less than 5%, imputation procedures were not used [19]. Descriptive statistics (mean, SD) and histogram displays were used to compare sociodemographic variables for the full sample, and the four independent groups defined by ICD indication and intervention condition. Oneway ANOVA was used (SPSS version 19.0) to describe baseline differences among four groups defined by (1) SCT Intervention or usual care) and (2) ICD indication: sudden cardiac arrest or other cardiac arrhythmia. The effects of ICD indication on intervention outcomes were compared using a two-factor, repeated measures design with generalized estimating equations (GEE), comparing changes from baseline to 12 months for indicators of physical function, psychological adjustment, and self-efficacy (*p* ≤ 0.05). The effect of ICD indication on intervention outcomes was examined across the 12-months post-ICD (Table 1), adjusting for the baseline values of outcome measures as well as age, gender, site, and ethnicity. These analyses were exploratory, designed to uncover potential differences associated with SCA, thus no adjustments were made for multiple tests [20]. Analyses were created post-hoc and thus results should be interpreted judiciously.

**Table 1:** Outcomes from hospital discharge to 12 months by intervention condition and ICD indication

Variables	SCA (mean ± SD)			Other Arrhythmia (mean ± SD)		
& Time	Intervention	Usual care	Total	Intervention	Usual care	Total
*Physical function*						
Symptoms (PCA)						
Discharge	12.03 ± 7.69	9.78 ± 6.07	11.09 ± 7.10	10.53 ± 6.83	9.91 ± 7.42	10.19 ± 7.13
1 mo	9.94 ± 7.17	10.63 ± 8.20	10.22 ± 7.54	7.87 ± 6.06	8.91 ± 7.48	8.42 ± 6.84
3 mo	9.11 ± 7.89	8.25 ± 8.14	8.76 ± 7.93	7.13 ± 7.47	8.42 ± 7.65	7.82 ± 7.56
6 mo	7.91 ± 6.00	7.09 ± 7.99	7.59 ± 6.80	6.37 ± 5.65	9.52 ± 7.57	8.04 ± 6.88
12 mo	7.03 ± 6.22	7.23 ± 8.55	7.11 ± 7.19	7.39 ± 6.99	9.58 ± 8.73	8.57 ± 8.01
Total	9.30 ± 7.20	8.67 ± 7.78	9.05 ± 7.43	7.87 ± 6.73	9.27 ± 7.74	8.62 ± 7.31
General physical health (PCS)						
Discharge	32.32 ± 8.30	35.35 ± 8.99	33.58 ± 8.66	33.40 ± 9.25	36.61 ± 9.57	35.14 ± 9.52
1 mo	37.82 ± 10.36	40.80 ± 8.15	39.01 ± 9.58	38.90 ± 9.84	41.68 ± 10.84	40.39 ± 10.43
3 mo	41.30 ± 10.16	44.68 ± 9.54	42.68 ± 9.97	41.67 ± 10.91	41.16 ± 11.33	41.40 ± 11.08
6 mo	42.65 ± 10.07	45.98 ± 7.98	43.96 ± 9.37	42.39 ± 10.55	42.58 ± 11.71	42.49 ± 11.13
12 mo	42.91 ± 10.11	42.81 ± 10.72	42.87 ± 10.26	40.72 ± 10.71	41.27 ± 12.25	41.01 ± 11.52
Total	39.19 ± 10.50	41.67 ± 9.75	40.19 ± 10.26	39.38 ± 10.68	40.60 ± 11.27	40.04 ± 11.01
ICD shocks						
Discharge	0.00 ± 0.00	0.04 ± 0.19	0.02 ± 0.12	0.00 ± 0.00	0.04 ± 0.19	0.02 ± 0.14
1 mo	0.03 ± 0.16	0.28 ± 0.68	0.13 ± 0.46	0.22 ± 0.81	0.50 ± 1.78	0.37 ± 1.42
3 mo	0.19 ± 0.74	0.04 ± 0.20	0.13 ± 0.59	0.13 ± 0.45	0.09 ± 0.35	0.11 ± 0.40
6 mo	0.14 ± 0.55	0.00 ± 0.00	0.08 ± 0.43	0.04 ± 0.21	0.58 ± 2.56	0.33 ± 1.88
12 mo	0.55 ± 1.48	0.13 ± 0.46	0.38 ± 1.18	0.20 ± 0.73	0.79 ± 1.65	0.52 ± 1.34
Total	0.17 ± 0.72	0.10 ± 0.39	0.14 ± 0.65	0.12 ± 0.44	0.37 ± 1.50	0.27 ± 1.04
*Psychological adjustment*						
Mental health (MCS)						
Discharge	52.17 ± 9.56	51.19 ± 9.38	51.76 ± 9.42	51.53 ± 9.88	52.67 ± 9.22	52.15 ± 9.50
1 mo	52.98 ± 7.54	51.39 ± 9.67	52.35 ± 8.41	53.52 ± 9.57	52.75 ± 8.76	53.11 ± 9.11
3 mo	54.00 ± 7.60	53.20 ± 9.04	53.67 ± 8.15	54.32 ± 9.66	52.87 ± 9.36	53.54 ± 9.48
6 mo	52.15 ± 9.11	55.35 ± 7.63	53.41 ± 8.63	55.06 ± 8.66	52.38 ± 8.85	53.64 ± 8.82
12 mo	54.30 ± 7.56	53.65 ± 8.30	54.03 ± 7.80	53.93 ± 9.24	52.71 ± 9.41	53.27 ± 9.30
Total	53.09 ± 8.30	52.86 ± 8.86	53.00 ± 8.52	53.66 ± 9.41	52.68 ± 9.06	53.13 ± 9.23
Anxiety (STAI)						
Discharge	36.76 ± 12.34	36.57 ± 11.23	36.68 ± 11.80	35.23 ± 11.66	31.71 ± 10.13	33.32 ± 10.94
1 mo	34.47 ± 10.52	33.21 ± 10.81	33.97 ± 10.56	32.80 ± 11.08	31.89 ± 10.15	32.31 ± 10.55
3 mo	32.86 ± 12.06	33.63 ± 11.29	33.17 ± 11.66	31.02 ± 11.52	33.00 ± 11.29	32.08 ± 11.38
6 mo	33.15 ± 11.91	30.27 ± 9.80	32.02 ± 11.13	30.11 ± 10.17	32.88 ± 11.80	31.58 ± 11.09
12 mo	31.34 ± 11.84	30.41 ± 10.53	30.96 ± 11.23	31.39 ± 12.03	32.19 ± 10.46	31.82 ± 11.15
Total	33.82 ± 11.76	32.99 ± 10.86	33.48 ± 11.39	32.13 ± 11.35	32.33 ± 10.71	32.24 ± 11.00
Depression (CES-D)						
Discharge	10.37 ± 7.82	10.85 ± 7.32	10.57 ± 7.56	11.81 ± 10.78	9.66 ± 7.55	10.64 ± 9.18
1 mo	9.64 ± 7.01	10.21 ± 9.08	9.87 ± 7.83	8.67 ± 8.63	8.23 ± 8.37	8.43 ± 8.45
3 mo	9.60 ± 9.50	8.67 ± 9.14	9.22 ± 9.29	8.74 ± 9.24	8.77 ± 8.78	8.76 ± 9.95
6 mo	8.00 ± 8.32	6.73 ± 7.46	7.50 ± 7.95	7.28 ± 7.07	9.50 ± 9.02	8.46 ± 8.20
12 mo	7.84 ± 8.22	9.00 ± 9.06	8.31 ± 8.51	8.27 ± 8.44	9.48 ± 8.37	8.93 ± 8.38
Total	9.14 ± 8.17	9.18 ± 8.41	9.16 ± 8.25	8.97 ± 8.98	9.13 ± 8.37	9.06 ± 8.65
*Self-efficacy*						
SCA self-efficacy expectations (SE)						
Discharge	8.04 ± 1.99	8.28 ± 1.64	8.14 ± 1.85	8.47 ± 1.70	8.98 ± 1.18	8.75 ± 1.45
1 mo	8.35 ± 1.65	8.75 ± 1.19	8.51 ± 1.48	8.88 ± 1.47	8.98 ± 1.35	8.93 ± 1.40
3 mo	8.74 ± 1.49	8.66 ± 1.56	8.71 ± 1.50	9.00 ± 1.32	8.95 ± 1.44	8.97 ± 1.38
6 mo	8.76 ± 1.48	8.65 ± 1.34	8.72 ± 1.41	9.12 ± 1.32	8.94 ± 1.63	9.03 ± 1.49
12 mo	8.98 ± 1.30	8.92 ± 1.71	8.95 ± 1.47	9.05 ± 1.31	9.08 ± 1.25	9.07 ± 1.27
Total	8.56 ± 1.63	8.64 ± 1.49	8.59 ± 1.57	8.90 ± 1.44	8.98 ± 1.37	8.95 ± 1.40
SCA self-management behavior (SMB)						
Discharge	7.80 ± 1.64	7.85 ± 2.15	7.82 ± 1.85	7.95 ± 2.13	8.61 ± 1.46	8.31 ± 1.82
1 mo	8.25 ± 1.76	8.72 ± 1.09	8.44 ± 1.54	8.77 ± 1.52	8.82 ± 1.37	8.80 ± 1.44
3 mo	8.78 ± 1.41	8.49 ± 1.61	8.66 ± 1.49	8.91 ± 1.42	9.03 ± 1.35	8.98 ± 1.37
6 mo	8.91 ± 1.15	9.00 ± 1.43	8.94 ± 1.26	8.99 ± 1.38	9.11 ± 1.21	9.05 ± 1.29
12 mo	9.04 ± 1.14	8.89 ± 1.74	8.98 ± 1.40	9.05 ± 1.34	9.09 ± 1.25	9.07 ± 1.29
Total	8.53 ± 1.51	8.56 ± 1.69	8.54 ± 1.58	8.73 ± 1.63	8.93 ± 1.34	8.84 ± 1.48
SCA outcome expectations (OE)						
Discharge	4.22 ± 0.47	4.38 ± 0.48	4.29 ± 0.47	4.29 ± 0.53	4.38 ± 0.49	4.34 ± 0.51
1 mo	4.24 ± 0.58	4.46 ± 0.50	4.33 ± 0.56	4.46 ± 0.47	4.31 ± 0.46	4.38 ± 0.47
3 mo	4.34 ± 0.58	4.51 ± 0.49	4.41 ± 0.55	4.44 ± 0.48	4.34 ± 0.48	4.39 ± 0.48
6 mo	4.42 ± 0.56	4.54 ± 0.45	4.47 ± 0.52	4.57 ± 0.49	4.39 ± 0.55	4.48 ± 0.53
12 mo	4.39 ± 0.63	4.48 ± 0.46	4.43 ± 0.57	4.55 ± 0.44	4.40 ± 0.44	4.47 ± 0.44
Total	4.32 ± 0.56	4.47 ± 0.47	4.38 ± 0.53	4.46 ± 0.49	4.37 ± 0.48	4.41 ± 0.49

Intervention condition = SCT Intervention versus Usual Care; ICD Indication = Sudden Cardiac Arrest versus Other Arrhythmias

## Results

### Demographic and characteristics

Characteristics of the intervention vs. usual care (UC) groups was reported previously [6, 7]. In brief, 243 patients were screened for study participation and 168 (69%) were randomized to either the SCT intervention (N = 85) or to usual care (N = 83). Of the 243 individuals who were screened, 28 (11.5%) were ineligible based on ventricular arrhythmia criteria; 8 (3.3%) did not return baseline questionnaires within 1 month of hospital discharge and were not enrolled; 18 (7.4%) chose not to continue after reviewing the questionnaire packet; 20 (8.2%) did not want to participate in the telephone intervention or could not be contacted after hospital discharge; and 1 person wanted to receive more money to participate. Of the 168 who were randomized over 12 months, 10 participants did not want to complete follow-up questionnaires; 2 developed terminal cancer and decided not to continue; 3 died from heart failure,1 due to renal failure, and 1 due to valvular heart disease; and 1 participant withdrew. Complete data were available on 150 of 168 (89%) at all time points over 12 months. There were no significant differences in baseline characteristics and outcomes for those who did vs did not complete all data collection. With two exceptions, there were no statistically significant differences between the study groups on baseline characteristics. Specifically, participants in the intervention group were more likely to live with a significant other or spouse with whom they had an intimate relationship (*p* = 0.01), and to have experienced a myocardial infarction (*p* = 0.05) prior to receiving the ICD.

Demographic and clinical characteristics between the SCA group (N = 65) vs the other arrhythmia group (N = 103) were examined post-randomization and are reported in Table 2. There were no statistically significant differences in baseline demographics between SCA and the Other Arrhythmia group. The Other Arrhythmia group included patients who received an ICD for VT or VF that was inducible or not at EPS testing (63%), sustained VT with syncope or pre-syncope (15%), unmonitored syncope with documented VT (13%), or VT lasting > 30 s (9%).

**Table 2:** Demographic and clinical characteristics

Variables	Sudden cardiac arrest	Other cardiac arrhythmia	*p*-value
	N = 65	N = 103	
*Sex*			
Male	48 (74%)	81 (79%)	0.47
Female	17 (26%)	22 (21%)	
Age (mean years)	63.5 ± 12.2	64.4 ± 12.4	0.64
BMI (mean kg/m^2^)	27.6 ± 5.6	28.8 ± 6.7	0.24
Charlson Co-morbidity Index	4.0 ± 2.3	4.6 ± 2.4	0.11
LVEF%	34.8 ± 15.3	33.1 ± 12.6	0.47
*Race*			
Caucasian	59 (91%)	91 (88%)	0.70
American Indian/Alaskan	1 (2%)	2 (2%)	
Asian/Pacific Islander	2 (3%)	2 (2%)	
Black/African American	3 (5%)	4 (4%)	
Mixed race/other	0 (0%)	3 (3%)	
*Education*			
Some high school or less	8 (12%)	13 (13%)	0.64
Graduated high school	11 (17%)	27 (26%)	
Some college	15 (23%)	25 (24%)	
Completed vocational program	2 (3%)	3 (3%)	
Completed 2 year college	9 (14%)	9 (9%)	
Graduated 4 year college	11 (17%)	10 (10%)	
Graduate degree	9 (14%)	16 (16%)	
*Employment status*			
Full-time	13 (20%)	24 (23%)	0.88
Part-time	8 (12%)	11 (11%)	
Not employed	4 (6%)	4 (4%)	
Retired	33 (51%)	55 (53%)	
Full-time housewife	1 (2%)	3 (3%)	
Disabled	6 (9%)	6 (6%)	
*ICD reason*			
VF sudden cardiac arrest	65 (100%)	0 (0%)	< 0.001
Sustained VT with pre-syncope/syncope	0 (0%)	16 (16%)	
Unmonitored syncope with Documented VT	0 (0%)	13 (13%)	
VT ≥ 30 s	0 (0%)	9 (9%)	
VT or VF inducible on EPS	0 (0%)	65 (63%)	
Myocardial Infarction	35 (54%)	61 (59%)	0.30
Diabetes Mellitus	17 (26%)	26 (25%)	0.52
COPD	6 (9%)	15 (15%)	0.22
Heart Failure	28 (43%)	51 (50%)	0.26
CVA	7 (11%)	20 (19%)	0.10
Smoker	13 (20%)	19 (18%)	0.48
Active alcohol use	2 (3%)	1 (1%)	0.33
Active opiate use	1 (2%)	1 (1%)	0.63

### Differences between the SCA and other arrhythmia groups over time

#### Physical function

Physical function indicators included the patient concerns assessment (PCA), general physical health (PCS), and ICD shocks (Table 3). For PCA symptoms, there was a statistically significant indication x time interaction (Wald X^2^ = 8.38, *p* = 0.04), showing that change over 12 months differed by SCA vs Other Arrhythmia. Patients in the SCA group compared to the Other Arrhythmia group reported more symptoms over 12 months, except at 12 months, when both groups reported similar symptom levels. There was a significant difference in the number of ICD shocks based on intervention and ICD indication (Wald X^2^ = 7.23, *p* = 0.007 for 2-way interaction), such that patients in the usual care Other Arrhythmia group received a higher number of ICD shocks. There was also a significant three-way interaction with intervention group described below.

**Table 3:** Summary of model effects for time, intervention group, ICD indication, and interactions

Wald X^2^, *p*-values for time, group, indication, and interaction effects							
Variables	Time	Group	Indication	Group x indication	Group x time	Indication x time	Group x indication x time
*Physical function*							
Symptoms PCA	**7.26, 0.06**	**3.85, 0.05**	0.12, 0.72	0.80, 0.37	2.00, 0.58	**8.38, 0.04**	3.52, 0.32
Physical Function PCS	**23.4, 0.0001**	0.09, 0.77	2.62, 0.11	1.36, 0.24	2.03, 0.57	3.45, 0.33	2.74, 0.43
ICD Shocks	**12.68, 0.005**	1.03, 0.31	3.04, 0.08	**7.23, 0.007**	6.52, 0.09	3.29, 0.35	**10.58, 0.01**
*Psychological adjustment*							
Mental Health (MCS)	1.64, 0.65	0.69, 0.41	0.07, 0.79	1.70, 0.19	1.36, 0.72	0.68, 0.88	**7.25, 0.06**
Anxiety (STAI)	3.43, 0.33	1.73, 0.19	1.05, 0.31	2.75, 0.10	1.74, 0.63	1.64, 0.65	2.61, 0.46
Depression (CES-D)	3.94, 0.27	1.23, 0.27	0.04, 0.84	2.17, 0.14	1.55, 0.67	2.24, 0.53	2.87, 0.41
*SCA self-efficacy*							
Self-Efficacy Expectations	**7.89, 0.05**	1.96, 0.16	0.05, 0.82	0.43, 0.51	4.38, 0.22	2.43, 0.49	0.87, 0.83
Self-management Behavior	**11.72, 0.008**	0.34, 0.56	0.08, 0.77	0.49, 0.48	3.79, 0.29	1.73, 0.63	4.23, 0.24
Outcome Expectations	**10.53, 0.015**	0.75, 0.39	0.52, 0.47	**6.99, 0.008**	0.84, 0.84	1.30, 0.73	0.57, 0.90

Group = Intervention versus Usual Care, Indication = sudden cardiac arrest versus other arrhythmia

Bold = significant interactions

#### Psychological adjustment

Indicators of psychological adjustment included the SF-36 Mental Composite Score (MCS), State Trait Anxiety Inventory (STAI), and Centers for Epidemiology Studies Depression (CES-D) scale. There were no statistically significant differences between SCA and the Other Arrhythmia group for outcomes of psychological adjustment (Table 3). For the MCS, the general U.S population mean is 50, with clinically significant change reported as 4 mean points [21]. Patients in this study reported mental health perception similar to the U.S. population. Using the STAI, scores of ≥ 30 reflect moderate anxiety, while scores of ≥ 40 reflect high anxiety [16]. Participants across all groups reported moderate levels of anxiety throughout the study. On average, study participants did not report clinically significant depression, indicated by CES-D scores ≥ 16 [17]. Depression scores were the highest at hospital discharge and declined across 12-months.

#### Self-efficacy

Self-efficacy indicators included measures of SCA self-efficacy expectations (SCA-SE), self-efficacy behavior (SCA-B), and SCA outcome expectations (SCA-OE). Across time, there were no significant differences in self-efficacy expectations, self-efficacy behaviors or outcome expectations between the SCA groups or the Other Arrhythmia group (Table 3).

### Differences between the intervention groups over time

#### Physical function

PCA symptoms showed differences by group (Wald X^2^ = 3.85, *p* = 0.05), indicating that those in the intervention group reported fewer symptoms compared to usual care over 12 months (Table 3). There were no significant intervention group differences noted by the PCS. Physical health scores ranged in the 30–40 s throughout 12 months, in general indicating perceptions of low physical health compared to the general U.S. population. Over time, PCS improved in the intervention group 8–10 mean points, whereas usual care improved an average of 5–7 mean points.

**Fig. 1:**
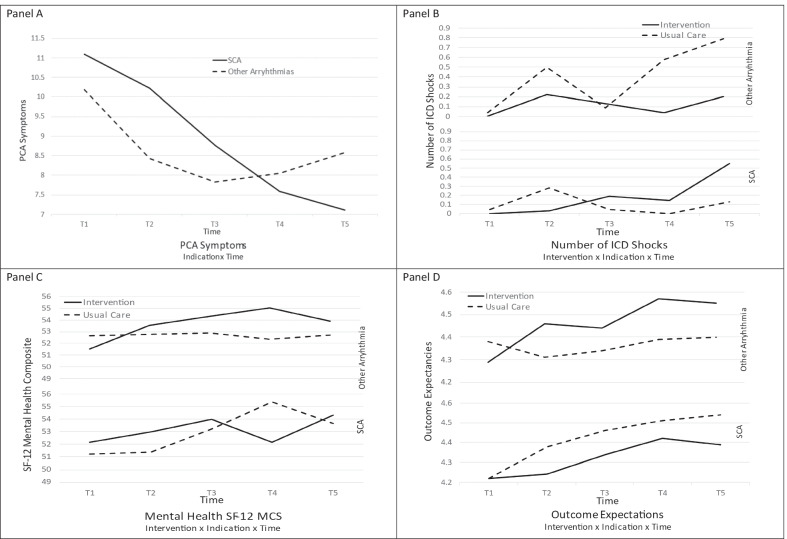
Summary of Model Effects for Time, Group, ICD Indication Interactions for PCA, ICD shocks, Mental Health (SF-12 MCS), Outcome Expectations

There was a significant intervention x time x indication interaction for ICD shocks (Wald *X*^2^ = 10.58, *p* = 0.01 for 3-way interaction, Table 3). Figure 1 shows that patients who had a SCA had a similar number of ICD shocks over 12 months, that the pattern of change differed significantly from the Other Arrythmia group, independent of the intervention group to which they were assigned. Those who received an ICD for Other Arrhythmias and participated in usual care received the highest number of shocks at 1, 6 and 12 months. Overall, however, few participants (< 5%) experience ICD shocks across the 12-month study period. The intervention was not expected to have a significant impact on the number of ICD shocks.

#### Psychological Adjustment

For general mental health (MCS), the intervention x time x indication interaction approached the statistical significance threshold (Table 3; Wald *X*^2^ = 7.26, *p* = 0.06). With the intervention, mental health gradually improved until 6 months for individuals in the Other Arrhythmia group, whereas those in usual care showed no change. SCA patients in usual care showed sharp improvement from the beginning of the study to 6 months, with a slight reduction in mental health by 12 months. In contrast, SCA patients in the intervention, showed declines in mental health (MCS) 3 to 6 months (post intervention), but recovered by 12 months.

#### Self-efficacy

There were no significant differences in self-efficacy expectations or behaviors between the two intervention groups. However, there was a statistically significant intervention x indication interaction effect for outcome expectations (Table 3; Wald *X*^2^ = 6.99, *p* = 0.01). Figure 1 illustrates that among participants in the Other Arrhythmia group, those in the intervention compared to usual care, consistently reported higher outcome expectancies throughout the 12 months. In contrast, for patients who had experienced SCA, those in the intervention compared to usual care, consistently reported lower outcome expectancies. Outcome expectations were higher across 12 months in those in the intervention who did not suffer SCA.

## Discussion

This study indicated that SCA survivors who received an initial ICD, compared to individuals who did not suffer a SCA but had an ICD implanted for a ventricular arrhythmia, had differing trajectories of recovery in physical function, psychological adjustment, and self-efficacy outcomes over 12 months while participating in a SCT intervention. The new findings from this analysis demonstrate that SCA survivors compared to patients with other arrhythmias had: (a) higher numbers of self-reported symptoms regardless of the intervention, (b) non-linear trajectory of recovery in self-reported mental health (decline in mental health at 6 months in intervention group, return to baseline by 12 months), with both indication groups ending up with similar mental health at 12 months, (c) lower numbers of ICD shocks over 12 months in the SCT intervention compared to those with other arrhythmias, and (d) lower outcome expectations over 12 months, but similar levels for both groups at 12 months.

Prior systematic reviews (N = 4) of QOL, physical, and psychosocial function following SCA have demonstrated there is significant heterogeneity in survivor characteristics, methodologies and measurement tools used, limiting conclusions that can be made of the impact. Elliott et al. [22] conducted a review of 70 studies of patient reported outcomes post-SCA, concluding that QOL after SCA was generally good in 46 studies, neutral in 17 studies, and poor in 7 studies. When survivors were asked to compare current QOL with the pre-SCA QOL, two studies [23, 24] noted that almost all judged they were happy with current QOL. Green et al. [25] reviewed cognitive function, QOL, and mental health post-SCA, concluding that fatigue is a common long term symptom, some survivors suffer from lack of independence in activities of daily living (ADLs) early in recovery, and mental health concerns are prevalent (61% experienced anxiety, 45% depression, 27% PTSD). Haydon et al. [26] reviewed 36 studies noting that QOL post-SCA was good, and did not differ markedly from the general population, ICU patients, or other survivors of cardiac disease. The 2 papers reporting QOL > 15 years post-SCA, demonstrated that QOL was acceptable longer term. This variability in measurement times and instruments has prompted suggestions that a more standard approach to assessment of patient centered outcomes following SCA be adopted [27].

In this study, those who had a SCA reported higher symptoms and fewer ICD shocks across 12-months. Higher levels of physical symptoms may be expected in SCA participants given they are recovering from resuscitation, ICU stays, and prolonged hospitalization. In addition, SCA participants reported lower physical health in the first month after the ICD implantation. However, from 3 to 12 months, SCA survivors reported improved physical health that became superior to the participants receiving an ICD for other cardiac arrhythmias.

Following hospital discharge, SCA survivors may experience a number of significant physical health impairments including musculoskeletal (MSK), neurological, and cognitive issues lasting weeks to months. Muscle weakness (7–17%), fatigue (50%), chest wall pain from CPR and procedures (4–66%), and speech or swallowing difficulty (0–11%) can be present for 3–12 months [1]. Physical and occupational therapy, exercise interventions, and other specific therapies have been shown to improve physical function. Education to patients and families about expectations post-arrest can improve adaptation to physical impairments [28].

Psychological adjustment was lower in SCA survivors in overall mental health, anxiety, and depression across the first 6 months. However, at 12 months, SCA survivors reached similar levels of psychological adjustment to those who did not have a SCA in all three psychological adjustment outcomes. The longest follow-up report in SCA survivors (up to 8 years) [29] noted that PTSD was present in 27%, with these individuals also reporting lower quality of life, self-care, and more pain and depressed mood. Those who were younger reported higher levels of PTSD. Dougherty [30] noted that anxiety, depression, anger, and stress was elevated in SCA survivors who received an ICD shock compared to those who had no ICD shock over the 1st year following resuscitation. Kamphius [31] followed SCA survivors over 12 months noting that significant anxiety was present in 61% at hospital discharge that was reduced to 49% over 12 months, depression was present in 37% and was not reduced at 12 months of follow-up. Comparing SCA survivors who received therapeutic hypothermia to those with STEMI after 6 months [32], SCA survivors had more frequent anxiety (24% vs. 13%) and more depression (19% vs. 8%). Factors related to more anxiety and depression included receiving ICD shocks, younger age, being female, and having more disease burden. Interventions used to address psychological recovery after SCA have included medications, cognitive behavioral therapy, self-management interventions, and psychotherapy [1].

Dimensions of self-efficacy had a similar trajectory independent of having a SCA. Self-confidence to care for oneself, self-management behaviors, and outcome expectations were similarly stable in all groups over time. Self-efficacy is the belief about one’s capabilities to exert control or self-confidence to perform a behavior, and is thought to be the most powerful causal determinant of motivation and performance [10]. Interventions to improve physical and psychological adjustment in post-ICD recovery have been developed, but self-efficacy, a potential mechanism of intervention effectiveness, has not often been studied as a mediator or an outcome of interventions. Evidence from RCTs that have tested the use of the SCT interventions demonstrate outcome benefits in a variety of chronic and cardiac conditions [33–36]. In our intervention research, we have demonstrated the important role of self-efficacy in impacting physical and psychological outcomes after an ICD [6, 7, 37]. In SCA survivors, strengthening elements of the intervention to impact self-efficacy and outcome expectations need to be considered.

Importantly, while resuscitation care for SCA has evolved over the last decade, little has changed in the post-SCA assessment, rehabilitation, and chronic illness management to address the longer term physical and psychological effects of survival. This SCT intervention was one of the first studies to implement a home-based telephone nursing intervention to impact health outcomes after ICD implant in this population [6, 7]. At the time the study was conducted, little was known about the trajectory of recovery in physical and mental of SCA survivors compared to all others who had a secondary indication for ICD implant. By determining the specific response to the SCT intervention in SCA survivors, we have uncovered unique aspects of recovery that are commonly impeded after SCA. The study provides clinically relevant knowledge for post-SCA supportive care that can be used to address these unique needs of SCA survivors, including recognition and management of symptoms, provision of more assessment and management of mental health issues, and increasing confidence that implementing self-care will improve one’s overall health.

### Strengths/limitations

There are decided strengths to this analysis. There was a relatively large number of SCA survivors who participated in the study over a short period of time. Data were collected prospectively so that changes and trends in outcomes could be described and reported. The SCT intervention was carried out with high fidelity, and there were few drop-outs over 12 months. Reliable and valid measures were used to represent each outcome of interest. The limitations of the analysis include the data were collected prior to the widespread use of therapeutic hypothermia post-resuscitation, thus certain outcomes (e.g. cognitive function) may not be similar to SCA survivors today. This RCT was conducted before the approval of ICD implantation for primary prevention of SCA. Thus, the generalizability of the findings are not applicable to persons who receive an ICD for primary prevention. The programming of ICDs for treating ventricular arrhythmias has changed since the original study was conducted, and thus may impact the total number of ICD shocks received by this study sample compared to patients managed with current ICD programming algorithms. The analysis was not powered to detect statistically significant differences between SCA patients and those with other cardiac arrhythmias, thus the study is exploratory and descriptive.

## Conclusions

Post-SCA care has changed little over the last several decades. However, because of improvements in EMS systems and post cardiac arrest resuscitation algorithms, there is a growing cohort of SCA survivors across the U.S. The study results demonstrate that SCA survivors have a different recovery trajectory following resuscitation and ICD implantation than do patients who receive an ICD for secondary prevention. Despite the SCT intervention, SCA survivors reported higher physical symptoms, lower levels of mental health and outcome expectations, and experienced fewer ICD shocks over 12 months. Future research should address the unique and special needs of SCA survivors in the early post-resuscitation period, as well as the longer term physical and mental health sequelae of SCA survival. Comprehensive assessment of physical and mental health at the time of hospital discharge should be instituted, so that important issues can be discovered and addressed to improve overall QOL in the aftermath of SCA. Following assessment, symptom management, psychological support, and rehabilitation interventions should be made available during the first year after SCA to facilitate recovery.

## Data Availability

The datasets used and/or analyzed during the current study are not publicly available because we are not finding a suitable repository, but are available from the corresponding author on request.

## Abbreviations

ANOVA: Analysis of variance
CES-D: Centers for epidemiologic studies- depression
EPS: Electrophysiological study
GEE: Generalized estimating equations
ICD: Implantable cardioverter defibrillator
IRB: Institutional review board
LVEF: Left ventricular ejection fraction
MCS: Mental composite score
NTS: Nursing telephone support
OE: Outcome expectations
PCA: Patient concerns assessment
PCS: Physical composite score
QOL: Quality of life
SCA-SE: Sudden cardiac arrest self-efficacy
SCA: Sudden cardiac arrest
SCT: Social cognitive theory
SI: Structured information
SMB: Self-management behaviors
STAI: State trait anxiety (STAI)
UC: Usual care
VF: Ventricular fibrillation
VT: Ventricular tachycardia
